# 30143 Asymptomatic Thoracic Aortic Aneurysm Growth Rates and Predicting Factors: A Systematic Review and Meta-Analysis

**DOI:** 10.1017/cts.2021.641

**Published:** 2021-03-31

**Authors:** Matthew Henry, Pieter Van Bakel, Mark MacEachern, Marion Hoffman Bowman, Kim Eagle, Himanshu Patel, Nicholas Burris

**Affiliations:** University of Michigan

## Abstract

ABSTRACT IMPACT: Through conducting this systematic review and meta-analysis, we will elucidate which factors influence thoracic aortic aneurysm growth, which will further help clinicians to properly stratify and manage their patients with TAAs. OBJECTIVES/GOALS: Thoracic aortic aneurysms (TAA) are an indolent but fatal disease, and the patient characteristics that predict both overall growth and growth rate are still not well characterized. Our goal is to conduct a systematic review and meta-analysis in other to better describe different patient characteristics that predict TAA growth. METHODS/STUDY POPULATION: M.M. conducted a search of Ovid MEDLINE, Embase, and Scopus to identify articles. Inclusion criteria were any longitudinal study reporting asymptomatic TAA growth, growth rates, or clinical proxies for growth such as dissection, rupture, emergency surgery, and death. M.H and P.B. independently applied the criteria to the results of the search. Conflicts were resolved by N.B. Data was extracted and risk of bias assessed independently by M.H. and P.B. Summary estimates of the outcome variables are combined across studies using standard meta-analysis methods. Heterogeneity is assessed via forest plots, chi2 test (Q test), and I2 statistic. Sensitivity analysis is conducted to assess robustness of the findings. RESULTS/ANTICIPATED RESULTS: The literature search resulted in 3,419 abstracts, of which 176 were included and thus require a full text review. Cohen’s Kappa coefficient was 0.64, indicating substantial agreement and high inter-rater reliability. We describe four categories of patient characteristics influencing the growth of asymptomatic TAAs: demographics, genetic or inheritable conditions, hemodynamic or biomechanical factors, and serum biomarkers. We describe the measure of effect for all variables. We anticipate there is a significant level of heterogeneity between studies, and potentially moderate risk of bias for many of the included studies as they are retrospective and observational in nature. Furthermore, we anticipate publication bias and evaluate it with funnel plots. DISCUSSION/SIGNIFICANCE OF FINDINGS: Understanding the factors that influence the growth of asymptomatic thoracic aortic aneurysms (TAA) is paramount, as the size and growth rate of TAAs dictates the clinical course. Little is understood about the degree to which different characteristics influence growth. This meta-analysis will help elucidate factors that promote TAA growth.

